# Epigenetic and Metabolic Changes in Root-Knot Nematode-Plant Interactions

**DOI:** 10.3390/ijms21207759

**Published:** 2020-10-20

**Authors:** Paola Leonetti, Sergio Molinari

**Affiliations:** Bari Unit, Institute for Sustainable Plant Protection (IPSP), Department of Biology, Agricultural and Food Sciences (DISBA), 70126 CNR Bari, Italy; paola.leonetti@ipsp.cnr.it

**Keywords:** antioxidant enzymes, DNA methylation, epigenetics, plant resistance, root-knot nematodes, ROS, tomato

## Abstract

Two wild-type field populations of root-knot nematodes (*Mi-Vfield, Mj-TunC2field*), and two isolates selected for virulence in laboratory on resistant tomato cultivars (*SM2V, SM11C2*), were used to induce a resistance reaction in tomato to the soil-borne parasites. Epigenetic and metabolic mechanisms of resistance were detected and compared with those occurring in partially or fully successful infections. The activated epigenetic mechanisms in plant resistance, as opposed to those activated in infected plants, were detected by analyzing the methylated status of total DNA, by ELISA methods, and the expression level of key genes involved in the methylation pathway, by qRT-PCR. DNA hypo-methylation and down-regulation of two methyl-transferase genes (*CMT2*, *DRM5*), characterized the only true resistant reaction obtained by inoculating the *Mi-1.2-*carrying resistant tomato cv Rossol with the avirulent field population *Mi-Vfield.* On the contrary, in the roots into which nematodes were allowed to develop and reproduce, total DNA was generally found to be hyper-methylated and methyl-transferase genes up-loaded. DNA hypo-methylation was considered to be the upstream mechanism that triggers the general gene over-expression observed in plant resistance. Gene silencing induced by nematodes may be obtained through DNA hyper-methylation and methyl-transferase gene activation. Plant resistance is also characterized by an inhibition of the anti-oxidant enzyme system and activation of the defense enzyme chitinase, as opposed to the activation of such a system and inhibition of the defense enzyme glucanase in roots infested by nematodes.

## 1. Introduction

Genetic natural resistance to root-knot nematodes (RKNs) is conferred in many plant species by a single dominant resistance gene (*R* gene) that specifically recognizes the proper avirulence (*Avr*) gene in the nematode. This ‘gene-for-gene’ recognition triggers the initiation of a cascade of defense responses, which ultimately lead to the halt of nematode development. Most of our information on the mechanisms underlying defense response of resistant plants upon a nematode attack is based on RKN-tomato interactions. A high number of genes (*Mi* series) have been identified in some clones of domesticated edible (*Lycopersicon esculentum L*.) and wild type (*L. peruvianum*) tomato [1]. However, the most diffused resistance gene that has been introduced in most commercial resistant tomato cultivars is *Mi-1.2*, conferring resistance against the three most diffused RKNspecies: *Meloidogyne incognita, M. javanica,* and *M. arenaria*. Resistance to specific isolates of the potato aphid, *Macrosiphum euphorbiae* [2] and to two biotypes of the white fly, *Bemisia tabaci* [3] is conferred by *Mi-1.2*, as well. *Mi-1.2* is known as the only *R*-gene conferring resistance against so different groups of parasites. A hypersensitive reaction (HR) is observed as an early expression of tomato resistance to RKNs; it consists of a prolonged oxidative burst caused by enhanced generation and cellular concentration of reactive oxygen species (ROS), which lead to a rapid and localized cell death and tissue necrosis. *Mi-1.2*-mediated resistance, as well as most *R*-gene-mediated defenses, relies on salicylic acid (SA)-dependent defense pathway [4,5,6,7]. SA over-production and spreading in root cells contribute to lesion formation and may cause the uncoupling and inhibition of electron transport detected in mitochondria extracted from roots of resistant tomato plants inoculated with RKNs [8]. Host resistance mechanisms implicate a thorough rearrangement of gene expression, which leads to the generation of an array of defense proteins involved in phytoalexin, lignin, proteinase inhibitors, and polyphenol biosynthesis [9]. Above all, immune reactions are always characterized by a high production of pathogenesis related- (PR-) proteins, which are the executioners of plant immunity [10].

The first step in plant innate immunity against pest attacks is a relatively unspecific response, a basal defense triggered by pathogen-associated molecular patterns (PAMPs), known as PAMPs triggered immunity (PTI). A nematode-associated molecular pattern (NAMP, NemaWater) was recently reported to be an activator of an early PTI response in plants correlated with hydrogen peroxide [11]. Cell-surface receptors known as NLR proteins (nucleotide binding domain, NDB, leucine-rich repeats, LRR) recognize such molecular patterns in the apoplastic spaces [12]. However, PTI can be overcome by adapted pathogens able to secrete effector molecules directly into the cells. RKNs are able to suppress the plant immune system through an array of effectors directly injected into the cells by their stylet and/or secreted from the cuticle in the root apoplasm [13,14,15]. This suppression leads to silencing or down-regulation of many defense genes in the attacked susceptible plants [16,17]. In *R*-gene carrying plants, however, specific effectors can be recognized by intracellular NLRs in the so-called effector-triggered immunity (ETI). Although nematode penetration is allowed in immunized plants, a deleterious reaction against nematodes is triggered when invading motile juveniles (J2) try to build up their feeding site. Specifically, about 12 h after root inoculation of *Mi-1.2-*carrying plants with incompatible J2, a rapid and localized cell death in tissues surrounding the nematode head could be observed [18].

Although signaling and transcription factors leading to genome rearrangement and gene up-regulation in plant disease resistance have widely been described [7,12,19], the link between disease resistance and DNA methylation has only recently been focused [20]. Biotic interactions can impact plant epigenetic configuration, which, in turn, regulates biotic interactions by modulating plant response [21]. Epigenetics studies the heritable changes in gene function that do not depend on DNA sequence, such as DNA methylation and de-methylation, chromatin rearrangements, and histone modification. DNA methylation consists in the addition of a methyl group to the cytosine bases of DNA to form 5-methyl-deoxy-cytosine. The amount of methylated DNA in plants is determined by de novo DNA methylation, methylation maintenance, and DNA de-methylation [22]. *De novo* methylation is catalyzed by domains rearranged methyl-transferases (DRM), whilst maintenance is performed by three classes of enzymes: the most predominant CG methylation by methyl-transferase 1 (Met1)*,* CHG methylation by chromo-methyl-transferases (CMT2 and CMT3), and CHH methylation by DRM2 or CMT2. The RNA-directed DNA methylation (RdDM) pathway promotes the sequence targeting by DRMs, through the synthesis of small-RNA (smRNA) [20].The activation of different types of epigenetic mechanisms upon nematode infection has extensively been reported [23]. Gene silencing, produced in successful development of nematodes on susceptible plants through the manipulation of phyto-hormone pathways, has been ascribed to the activation of smRNA and miRNA pathways [16,24]. 

In this study, a screening of the possible reactions of different genotypes of tomato to different species and pathotypes of RKNs has first been carried out. Plant resistance to RKNs is rarely expressed as a total immunity to the parasites, rather, as a variable level of nematode development and reproduction restriction, according to the tested specific plant–nematode interactions. Therefore, we used wild-type populations belonging to two different RKN species, *M. incognita* and *M. javanica.* From both these field populations, a selection for virulence was carried out to obtain genetically homologous virulent isolates. Infections of resistant and susceptible tomato genotypes were provoked by means of these four different RKN genotypes. Different levels of parasitism could be compared and studied, from full to partial resistance and susceptibility. Resistance-breaking nematode populations are increasingly being found in extensive crop cultivations and considered as an actual threat to sustainable Integrated Pest Management (IPM) strategies [25]. Therefore, efforts should be spent to investigate the mechanisms of a resistance to RKNs that must be not only be effective but also durable. We also used virulent isolates to study, at epigenetic and biochemical level, how such pathotypes may be able to develop, although partially, on resistant genotypes. Moreover, the objective was to determine if full and partial resistance, or full and partial compatibility, were characterized by the same epigenetic and metabolic events. To do so, we compared the epigenetic and metabolic changes occurring in susceptible and field resistant tomato plants challenged by either RKN field populations or selected isolates. Generally, the most consistent epigenetic and metabolic changes seemed mainly to occur in plants attacked by wild-type field nematode populations causing full resistance and full susceptibility.

## 2. Results and Discussion

### 2.1. Resistance and Susceptibilityof Tomato to RKNs

**Figure 1:**
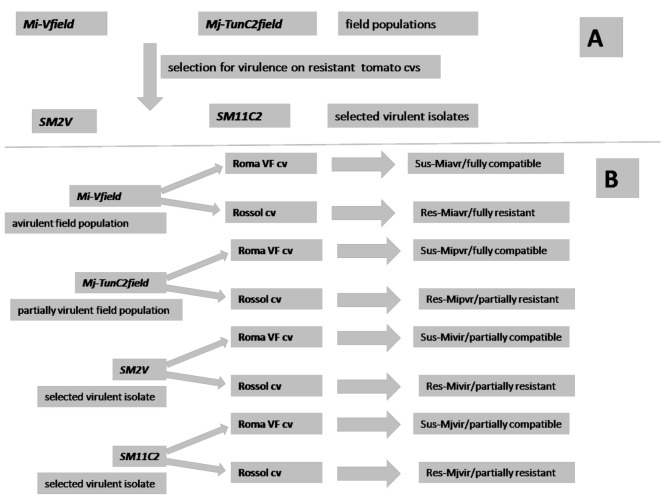
(**A**) Scheme of the selection for virulence to which the field populations *Mi-Vfield* and *Mj-TunC2field* were subjected to obtain the virulent isolated *SM2V* and *SM11C2*; (**B**) scheme of the nematode-plant interactions realized in this study.

Plants are defined as resistant when the attacking nematodes show reduced levels of reproduction [26]. Only when reproduction is as low as 25–50% of that on a fully susceptible cultivar, tomato plants are generally considered as slightly resistant to RKNs; fully resistant plants usually support a reproduction lower than 10%.Avirulent wild-type populations, when subjected in nature to repeated exposures to *R*-genes, may generate resistance-breaking virulent populations. This “natural” selection can be mimicked in controlled greenhouse conditions by, at first, collecting the few egg masses developed on *Mi-1*-carrying tomato by wild-type field populations; repeated inoculation of this selected progeny leads to the generation of stable virulent isolates [27]. This procedure was repeated in this study so that it was possible to produce from the wild-type populations *Mi-Vfield*, collected from Venezuela, and *Mj-TunC2field,* collected from Tunisia, the virulent isolates *SM2V* and *SM11C2*, respectively, that reached their full reproductive potential within the second/third generation (Figure 1A). *Mj-TunC2field* initially showed a more consistent development on resistant tomato than many other tested field populations. Therefore, it was considered as a "partially virulent natural population" and the response of the resistant cultivar Rossol only as "partially resistant" (Figure 1B). It is already known that some RKN populations exist in tropical or subtropical countries, commonly referred to as “natural virulent” that may or may not have had a previous and not documented exposure to *Mi-1* gene or analogues. Using 2 field populations and their selected virulent isolates to test resistant and susceptible tomato plants, we produced both partially and fully resistant and susceptible responses. Thus, we were able to compare the epigenetic and metabolic changes associated with four different types of plant response to nematodes (Figure 1B). 

**Figure 2:**
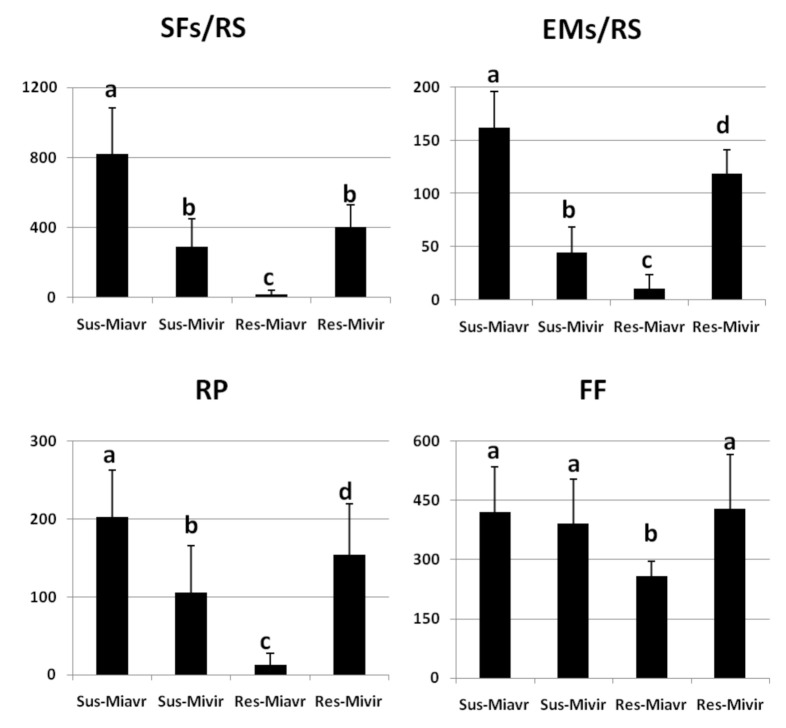
Infection factors of susceptible and resistant tomato plants inoculated with the avirulent *Meloidogyne incognita* field population *Mi-Vfield* and the selected virulent isolate *SM2V*. Infection factors of four interactions are shown: Roma VF/*Mi-Vfield* (Sus-Miavr); Roma VF/*SM2V* (Sus-Mivir); Rossol/*Mi-Vfield* (Res-Miavr); Rossol/*SM2V* (Res-Mivir). Infection level was characterized by the numbers of Sedentary Forms per Root System (SFs/RS), Egg Masses per Root System (EMs/RS), Reproduction Potential (RP), and Female Fecundity (FF).Values are expressed as means (*n* = 9) ± standard deviations. Means were separated by a Duncan’s Test, different letters indicate significantly different means (Significance Level: 0.05).

**Figure 3:**
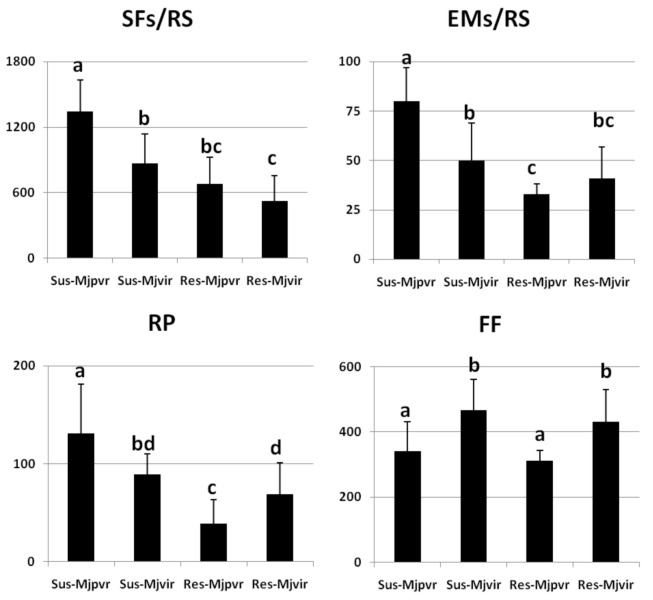
Infection factors of susceptible and resistant tomato plants inoculated with the partially virulent *Meloidogyne javanica* field population *Mj-Tunc2field* and the selected virulent isolate *SM11C2*. Infection factors of four interactions are shown: Roma VF/*Mj-TunC2field* (Sus-Mjpvr); Roma VF/*SM11C2* (Sus-Mjvir); Rossol/*Mj-TunC2field* (Res-Mjpvr); Rossol/*SM11C2* (Res-Mjvir). Infection level was characterized by the numbers of Sedentary Forms per Root System (SFs/RS), Egg Masses per Root System (EMs/RS), Reproduction Potential (RP), and Female Fecundity (FF). Values are expressed as means (*n* = 9) ± standard deviations. Means were separated by a Duncan’s Test (Significance Level: 0.05).

We tested these RKN populations/isolates in their ability to produce sedentary forms and egg masses per root system (SFs-EMs/RS), reproduction potential (RP), and female fecundity (FF) as a result of their inoculation on resistant and susceptible tomato plants (Figure 2 and Figure 3).

EMs/RS, RP, and FF are indices of the reproductive rate of a given nematode population reproducing on a determined plant genotype. However, unsuitable nematode-plant interaction assays, an index of the actual disease severity, and plant damage must be given. Root gall index (GI) has commonly been used so far to determine disease severity caused by RKNs to plants, since the amount of galls determines the impairment of nutrient and water transports along the plant, thus causing poor shoot growth and yield loss. However, GI is detected by a direct visual analysis of the roots by the operator and can presumably state if a plant has been infested or not; however, it may represent an unreliable indicator when intermediate degrees of galling must be distinguished, as it occurs in partial resistance or susceptibility [28]. Therefore, we used the number of developed sedentary individuals extracted from roots (SFs/RS) as a more quantitative factor of root damage and gall index, since galling is a reaction to motile individuals that enter the roots, establish a feeding site, and turn into sedentary developmental stages [27].

The avirulent field population *Mi-Vfield* showed a high RP and high numbers of SFs/RS on susceptible tomato (approx. 200 and 800, respectively) but very low ones (approx. 13 and 17) on resistant tomato, as expected (Figure 2). The ability to develop and reproduce on resistant tomato drastically increased in the virulent isolate *SM2V.* The partial virulent wild-type population *Mj-TunC2field* was very aggressive on susceptible tomato with more than 1200 SFs/RS (Figure 3). Increased competition for food lessened both FF and RP of *Mj-TunC2field*, with respect to those of *Mi-Vfield*. Of course, *Mj-TunC2field* was much less aggressive on resistant tomato, showing a relatively low RP value. The virulent isolate *SM11C2*developed on resistant tomato at a level similar to its starting field population *Mj-TunC2field*; conversely, because of its higher FF value, its overall reproductive rate resulted higher. The selective pressure operated on both isolates and the resulting genetic homogeneity may have been the cause of the observed diminished aggressiveness and reproduction rate on susceptible plants, with respect to the starting field populations (Figure 2 and Figure 3). *Mi-1.2-*conferred resistance was broken by those virulent isolates, although they could not achieve the reproduction rates observed in full compatibility. Actually, full compatibility was realized only in the interactions between field populations and susceptible tomato, whilst there was full incompatibility in the interaction between *Mi-Vfield* and Rossol (Figure 1B). 

### 2.2. DNA Methylation in Resistance and Susceptibility of Tomato to RKNs

Generally, resistance reaction, HR, and plant priming against RKNs are associated with a diffused up-regulation of defense genes, and, in particular, of pathogenesis-related genes (PR-genes) [6,17,29,30]; in contrast, susceptibility is characterized by PR-gene down-regulation and SA-signaling inhibition, although some genes, encoding for anti-oxidant enzymes, were found to be activated [17].

Epigenetic changes were shown to regulate the expression of genes involved in plant resistance response [20]. DNA methylation, besides many other biological processes, is involved in mechanisms underlying plant response to pathogen and nematode attacks [20,23]. Methylation on promoter regions negatively correlates with gene expression levels [31], whereas DNA hypo-methylation has been associated with plant defense against nematodes [23]. Hypo-methylation of resistance genes enhances their expression to rapidly respond to environmental factors [20].

**Figure 4:**
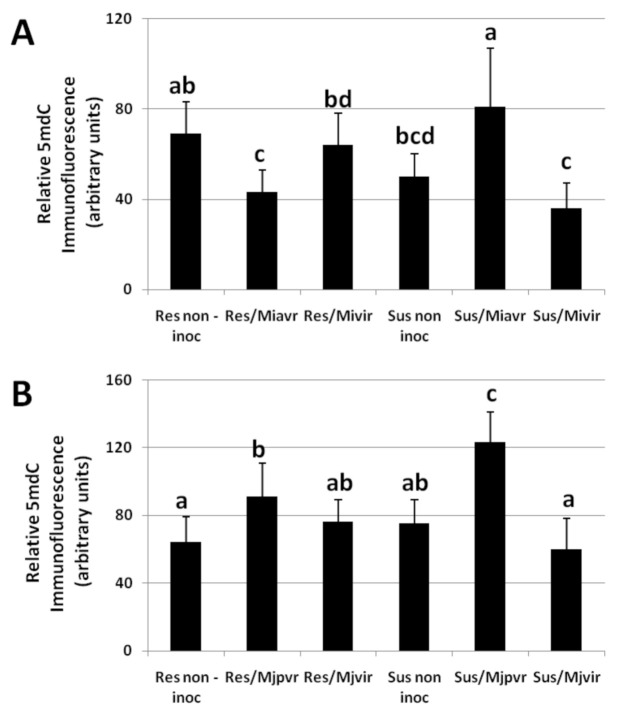
Arbitrary units of methylation on total DNA in roots of susceptible and resistant tomato plants un-inoculated or inoculated with 2 couples of field populations/virulent isolates of RKNs. Data were taken at 7 DAI. DNA was extracted from resistant (Res non-inoc, Rossol) and susceptible (Sus non-inoc, Roma VF) un-inoculated plants. In **A**, DNA was also extracted from roots of Rossol and Roma VF plants inoculated with the avirulent *Meloidogyne incognita* field population *Mi-Vfield* and the selected virulent isolate *SM2V*: Rossol/*Mi-Vfield* (Res-Miavr); Rossol/*SM2V* (Res-Mivir); Roma VF/*Mi-Vfield* (Sus-Miavr); Roma VF/*SM2V* (Sus-Mivir). In **B**, DNA was also extracted from roots of Rossol and Roma VF plants inoculated with the partially virulent *Meloidogyne javanica* field population *Mj-Tunc2field* and the selected virulent isolate*SM11C2*: Rossol/*Mj-TunC2field* (Res-Mjpvr); Rossol/*SM11C2* (Res-Mjvir); Roma VF/*Mj-TunC2field* (Sus-Mjpvr); Roma VF/*SM11C2* (Sus-Mjvir). Values are expressed as arbitrary units of relative 5mdC immunofluorescence and as means (*n* = 6) ± SD. Means were separated by a Duncan’s Test (Significance Level: 0.05).

Relative 5-mdC immunofluorescence, indicating the methylation state of total DNA, was detected in roots collected from a series of compatible and incompatible tomato-RKN interactions, at seven days after inoculation (DAI) (Figure 4). A significant hypo-methylation was observed in roots from resistant tomato infected by the wild-type population *Mi-Vfield* (Res/Miavr), whereas hyper-methylation characterized fully successful nematode infections (Sus/Miavr, Sus/Mjpvr), with respect to uninfected roots (Figure 4A,B). Interestingly, infections by virulent isolates to both resistant and susceptible plants (Res-Sus/Mivir, Res-Sus/Mjvir) did not produce significant changes in DNA methylation of roots, with respect to uninfected controls (Figure 4). These data show that total DNA hypo-methylation is associated with enhanced defense gene expression in immune response, triggered by R-genes (ETI) against RKNs. Comparably, DNA hypo-methylation has already been reported to be part of a conserved PTI response to nematodes in monocot and dicot plants [23]. In contrast, gene silencing, induced by nematodes in host plants, might result from the hyper-methylation of total DNA observed in the present study in successfully infected susceptible plants. Unfortunately, no data exist on changes in defense gene expression of roots attacked by selected virulent isolates. Surely, infections caused by such isolates on host plants (partially compatible interactions)are much less severe than the ones provoked by the populations collected in fields; moreover, although these selected isolates are able to develop also on resistant plants (partially incompatible interactions), their development in our experiments was proven to be generally limited. In these intermediate responses to RKNs, we were not able to detect significant changes in DNA methylation of plant roots. Therefore, DNA hypo-and hyper-methylation could be observed only in full resistance and full susceptibility, respectively.

### 2.3. Expression of Genes Involved in DNA Methylation

**Figure 5:**
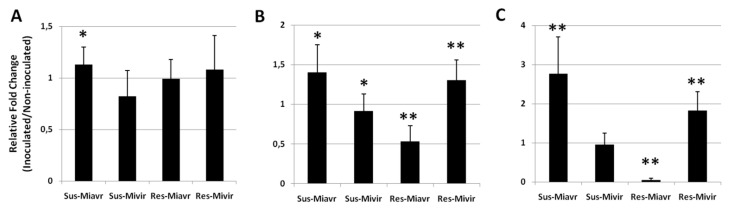
Expression of *Met1* (**A**)*, CMT2* (**B**), and *DRM 5* (**C**) genes in susceptible (Sus) and resistant (Res) tomato roots inoculated with the RKN field population *Mi-Vfield* (Miavr) and the virulent isolate *SM2V* (Mivir). Gene expression was detected at 7 DAI by quantitative real-time reverse-transcription polymerase chain reaction (qRT-PCR). Data are the mean fold changes (*n* = 6) ± SD in gene transcript levels of tissues from inoculated plants compared with tissues from non-inoculated control plants (the value of 1 indicates no change). Asterisks indicate that the mean fold change is significantly different from 1 as determined by a *t* test (*p < 0.05; **p < 0.01).

We detected the expression of *methyl-transferase 1* (*Met1,* A)*, chromo-methyl-transferase 2*(*CMT2*,B)*,* and *domains rearranged methyl-transferase 5* (*DRM5,*C) genes in roots of resistant (Res) and susceptible (Sus) plants, infected by the avirulent field population *Mi-Vfield* (Miavr) and the selected virulent isolate *SM2V* (Mivir); then, the expressions of these genes were compared with the ones from uninfected roots (Figure 5). The most interesting result was the drastic repression of *DRM5* and the halving of the *CMT2* transcript amounts in the incompatible interaction with respect to the uninfected resistant roots (Res-Miavr, Figure 5B, C). The reported DNA hypo-methylation in this same interaction can probably be associated with the suppression of *de novo* methylation and the inhibition of methylation maintenance. On the contrary, these genes had their expression enhanced in all the tested partially (Sus/Res-Mivir) and fully (Sus-Miavr) compatible interactions. Moreover, in the full compatible interaction Sus-Miavr, also the*Met1* gene was up-regulated (Figure 5A). The up-regulation of all the tested DNA methyl-transferases in fully infected roots, with respect to uninfected roots, supports the finding of a high DNA methylation state in such types of interactions with a possible consequent gene silencing.

### 2.4. Metabolic Mechanisms in Resistance and Susceptibility of Tomato to RKNs

**Figure 6:**
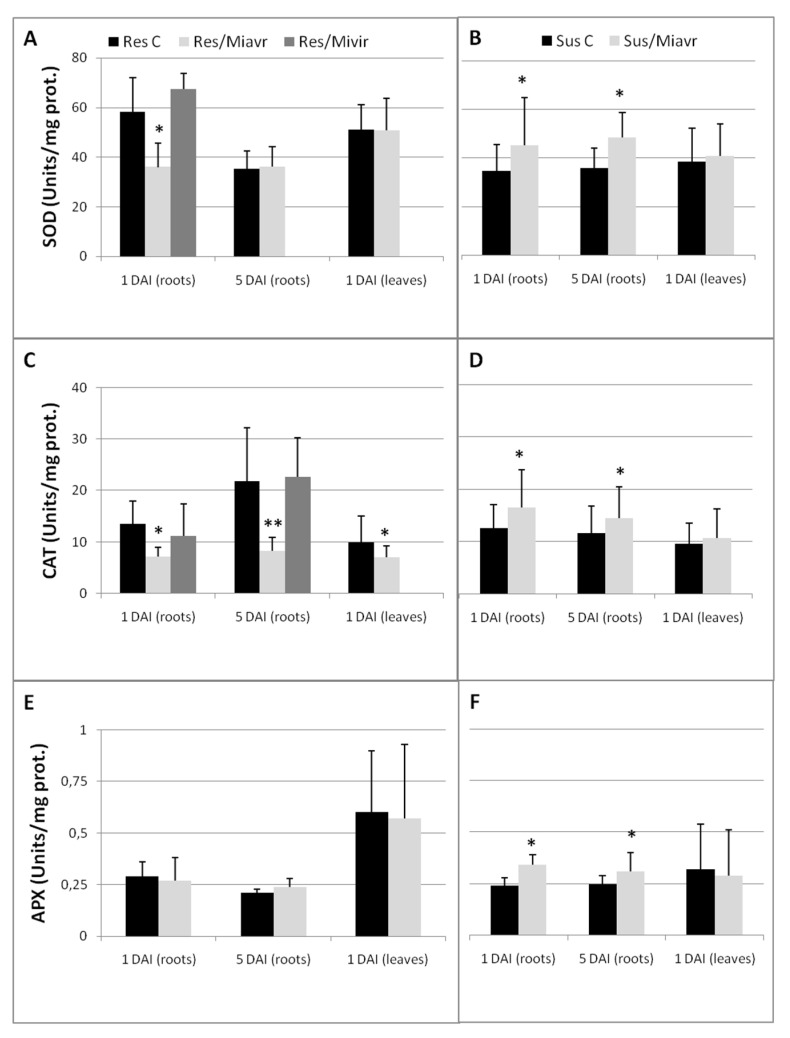
Anti-oxidant enzyme activities of resistant (Res C) and susceptible (Sus C) un-inoculated control plants compared with those of plants inoculated with the avirulent field population *Mi-Vfield* (Res/-Sus/Miavr) and with the virulent isolate *SM2V* (Res/Mivir). Superoxide dismutase (SOD—**A**,**B**), catalase (CAT—**C**,**D**), and ascorbate peroxidase (APX—**E**,**F**) activities are expressed as Units mg^-1^ prot. Protein extraction was carried out using roots 1 and 5 DAI and using leaves 1 DAI. Values are shown as means (*n* = 9) ± SD. Means coming from tissues of inoculated plants were separated from those of un-inoculated controls by a *t*-test; asterisks indicate significant difference (*p < 0.05; **p < 0.01).

Catalase activity (CAT) changes seem to be strictly associated with metabolic response to RKNs in tomato. Inhibition of CAT was found to occur in resistance response, whilst CAT was enhanced in successful infections (Figure 6C, D). CAT inhibition was found also in leaves of inoculated resistant plants as early as 1 DAI. SA is a known inhibitor of root and leaf CAT [32]. SA was found to be overproduced in roots and shoots of resistant tomato attacked by *M. incognita* [33]. Actually, most of the SA produced in roots may rapidly be transferred to leaves, in analogy with the SA adsorbed by roots from a solution [32]. SA is the main mediator for systemic acquired resistance (SAR), which provides long-term resistance to hemi-biotrophic pathogens and pests, and is correlated to the activation of PR-genes [17]. SAR has been reported to occur in upper leaves, in response to the attacks of pathogens that cause cell death and tissue necrosis at lower leaves [34]. RKNs actually produce cell death and tissue necrosis in roots of resistant plants as a result of a HR, which is most likely caused by an elevated concentration of SA and potentiation of ROS generation [35]. Over-expression of *PR*-genes in leaves of *Mi-1.2-*carrying tomato plants has been reported 5 DAI with *M. incognita*; thus, induction of SAR in leaves is likely to be the result of SA generation in roots and its movement upwards [6]. Successful defense reaction can be explicated only if ROS level is maintained high by ROS overproduction and inhibition of ROS-scavenging activities. In addition to CAT, Superoxide dismutase (SOD) was also inhibited at the earliest stage of an incompatible interaction, whilst ascorbate peroxidase (APX) restriction seems not to be involved in the resistance response (Figure 6A,E). Conversely, successful infection required high activities of anti-oxidant enzymes in roots (Figure 6B,D,F). 

High CAT activity may result from the reported up-regulation of CAT gene in compatible tomato-RKN interactions, which was analogously observed only in infected roots [17]. Activation of anti-oxidant enzymes, induced by nematodes in the host plants, is functional for their protection from the ROS typically generated in the early response to biotic challenges. For instance, RKNs have been reported to secrete an effector (MjTTL5), which interacts directly with another anti-oxidant enzyme in *Arabidopsis*, theferrodoxin: thioredoxin reductase catalytic subunit (FTRc), to induce an enhanced ROS-scavenging activity [36]. 

**Figure 7:**
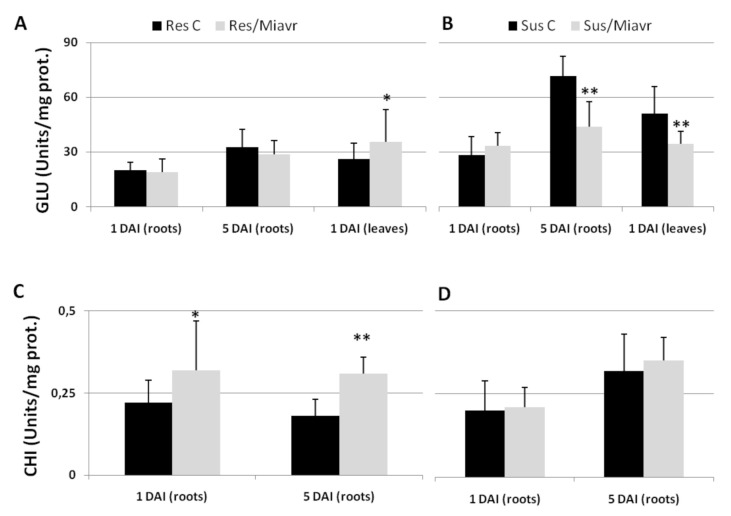
Defense enzyme activities of resistant (Res C) and susceptible (Sus C) un-inoculated control plants and of plants inoculated with the avirulent field population *Mi-Vfield* (Res/-Sus/Miavr) or with the virulent isolate *SM2V* (Res/Mivir). Glucanase (GLU—**A**,**B**), and chitinase (CHI—**C**,**D**) activities are expressed as Units mg^-1^ prot. Protein extraction was carried out using roots 1 and 5 DAI and using leaves 1 DAI, as it concerned GLU; CHI was assayed only in roots 1 and 5 DAI. Values are shown as means (*n* = 9) ± SD. Means coming from the inoculated roots were separated from those of un-inoculated control roots by a *t*-test; asterisks indicate significant difference (*p < 0.05; **p < 0.01).

β-1,3-Endoglucanase (GLU) and chitinase (CHI) are defense-induced enzymes in plants. GLU increased in leaves of infested resistant plants at 1 DAI, with respect to uninfected plants, whilst GLU was left unaffected in roots (Figure 7A). Conversely, CHI was found to enhance in roots of infested resistant plants (Figure 7C). Activation of CHI and over-expression of its encoding gene *PR-3* have already been reported in genetic and induced plant resistance to RKNs [17,37]. On the contrary, in susceptible tomato-*M. incognita* interaction, GLU resulted inhibited in the roots (5 DAI) and leaves (1 DAI) of infested plants (Figure 7B). No change in CHI was detected (Figure 7D). 

Accordingly, *PR-2*, the gene encoding for endoglucanases, was found to be down-loaded in roots of susceptible tomato plants at 5 DAI with *M. incognita*, but over-expressed in leaves of inoculated resistant plants [6]. 

### 2.5. Concluding Remarks

Epigenetic changes in total DNA methylation are being recognized of paramount importance in determining the outcome of nematode-plant interactions. DNA hypo-methylation, probably caused by the inhibition of methyl-transferase gene expressions, seems to be associated with the enhanced expression of defense genes in PTI and ETI of plants against RKNs. On the contrary, gene silencing in nematode heavily infected plants is likely to be preceded by DNA hyper-methylation and activation of methyl-transferase gene expressions. Interestingly, in infections caused by genetically homogeneous selected isolates to both susceptible or resistant plants, which were characterized by a less consistent numbers of galls, DNA methylation general status of roots seems not to be enhanced, although the expression of some methyl-transferase genes was found to be up-regulated. 

It is now generally recognized that plant epigenome can influence plant phenotype and biotic interactions. However, soil-borne sedentary endo-parasites, such as RKNs, can in turn induce epigenetic changes to address plant metabolism toward the most suitable conditions for their own development. Genetic resistance does not allow RKNs to impair plant immune reaction by rapidly producing PR-proteins and ROS, toxic to the invading J2. The augmented defense gene expression observed in plant immunity seems to be supported by a hypo-methylation of total DNA with respect to unchallenged plants. Moreover, the general process of plant priming against pests, diseases, and abiotic stresses may be based on similar epigenetic modifications that suppress or enhance the transcription of key regulators of the immune system. These environment-induced epigenetic changes may be transmitted to next generations for acclimation to a changing environment. Additional research on the relationship between epigenetics and biotic interactions should be supported for information on plant adaptation and crop improvement to face the increasing emergence of local or alien pests, also in view of the climate changes we are experiencing. The knowledge of such mechanisms would lead to the arrangements of environmental-friendly strategies for sustainable protection against biotic challenges based on the long-lasting immune memory in plants.

## 3. Materials and Methods

### 3.1. Nematode Populations

Two virulent isolates (*SM2V, SM11C2*) were selected from 2 field populations (*Mi-Vfield* and *Mj*-*TunC2field*, respectively) by repeated mass inoculation on *Mi-*carrying resistant tomato cvs, as described in [38]. *Mi-Vfield*and*Mj-TunC2field*were collected from infested plants located in fields in Venezuela and Tunisia, respectively, and maintained on susceptible tomato in a glasshouse. Nematodes were species identified as *M. incognita* (*Mi-Vfield*) and *M. javanica* (*Mj-TunC2field*) by means of isozyme electrophoretic patterns of esterase and malate dehydrogenase. *Mi-Vfield* had an initial negligible reproduction on resistant tomato, therefore it was classified as “avirulent” field population; in contrast, *Mj-TunC2field* had an initial consistent reproduction on resistant tomato and was classified as “natural partially virulent field population”. A higher number of repeated inoculations on resistant tomato occurred to select *SM2V* from *Mi-Vfield* than those needed to select *SM11C2* from *Mj-TunC2field*. Selection was considered to be completed when the selected isolate reached a reproduction rate on resistant tomato that could not significantly be exceeded by the next generation.

### 3.2. Preparation of Plants and Nematode Inoculations

Seedlings of the cv Roma VF were used as the tomato line susceptible to root-knot nematodes (RKNs), whilst the *Mi-1.2-*carrying resistant cvs used were Motelle, VFN8, and Rossol [39]. All resistant cvs were used to select virulent isolates from RKN field populations. Rossol was used as the tomato line resistant to RKNs in all experiments. After surface sterilization, seeds of Roma VF and Rossol cvs were sown in a sterilized mixture of peat and soil at 23–25 °C. Seedlings were transferred to clay pots (100 cm^3^ in volume) which were filled with a sterilized mixture of loamy soil and sand (1+1 by volume). Temperature-controlled benches, located in a glasshouse, were used to maintain at 24–25 °C the soil temperature of pots, which were randomly disposed. A regular regime of 12 h light/day was set, and plants were regularly watered with Hoagland’s solution. Plants, before being inoculated with nematodes, were allowed to grow to the 3–5 compound leaves stage and to 2–4 g fresh weight. Field nematode populations and their respective virulent isolates were used to inoculate both susceptible and resistant plants. Eight different tomato-RKN interactions were then analyzed and named as follows(see Figure 1):(1) Roma VF/*Mi-Vfield* (Sus-Miavr); (2) Roma VF/*SM2V* (Sus-Mivir); (3) Rossol/*Mi-Vfield* (Res-Miavr); (4) Rossol/*SM2V* (Res-Mivir); (5) Roma VF/*Mj-TunC2field* (Sus-Mjpvr); (6) Roma VF/*SM11C2* (Sus-Mjvir); (7) Rossol/ *Mj-TunC2field* (Res-Mjpvr); (8) Rossol/*SM11C2* (Res-Mjvir). Inoculations were carried out by pouring, into 2 holes made at the base of each plant, a few milliliters of a stirring water suspension containing 250 active J2. J2 used for inoculation were obtained by incubation of the respective egg masses in tap water at 27 °C for 2–3 days. 

### 3.3. Tests of Tomato Resistance and Susceptibility to RKNs

Plants were harvested approximately 7 weeks after inoculation to let nematodes complete their lifecycle and plants be infested by the second generation. Roots were cut from shoots and washed free of soil debris. Weights of roots and shoots were measured. Two root systems of plants from the same interaction were chopped into pieces of about 2 cm length and accurately mixed to be used for nematode life-stage extractions and counting. Three different samples of about 2 g were separated from the mix to be used for: (i) egg masses (EMs) counting; (ii) eggs extraction; iii) developed sedentary forms (SFs) extraction. For EMs counting, the gelatinous masses were red-colored by immersion of root samples in 0.1 g L^−1^ Eosin Yellow and stored in a refrigerator for at least 1 h. Samples were scored for red-colored egg masses under a stereoscope (6×magnification). Extraction of sedentary forms (J3, J4, swollen females) from roots was preceded by an incubation in a mixture of the enzymes pectinase and cellulase at 37 °C in an orbital shaker to loosen the bindings between sedentary nematodes and roots. Afterwards, roots were ground in physiological solution and the sedentary forms collected on a 90 µm sieve. Aliquots (2 mL) of stirring nematode suspensions were pipetted in small petri dishes and the numbers of SFs counted under a stereoscope (12×magnification). Eggs were extracted by sodium hypochloride according to the protocol described in [40]. Eggs suspensions were counted under a stereoscope at 25×magnification. These calculations produced values of EMs, eggs, and SFs per root system (RS) for the eight tested nematode-tomato interactions. Two additional infection factors were determined as follows:

1. Reproduction Potential = n. eggs (root system)/n. inoculated J2 (RP); this factor indicates the number of times the initial population (Pi) multiplies at the end of the experimental time (Pf). RP is particularly important to predict the population density to which the next crop will be exposed.
2. Female Fecundity = n. eggs (root system)/n. EMs (root system) (FF); it indicates the average number of eggs laid by a single female.

In the experimental conditions adopted in this study, only the inoculated J2 can reach the reproductive stage (gravid females producing eggs embedded in EMs). The juveniles hatched in pots from these eggs can develop into sedentary forms, but cannot reach the reproductive stage. This is why SFs/RS can exceed the one thousand units as compared with the 250 J2 inoculated per plant in a fully compatible interaction (field populations versus susceptible tomato). Furthermore, approximately 50% of the inoculated J2 reach the reproductive stage in a fully compatible interaction. On the other hand, when a plant-nematode interaction produces a RP 25–50% lower than that from a fully compatible interaction, a partial resistance response can be predicted. Actually, values of EMs, RP, and FF are indicative of the infection level caused by the first generation produced by the artificial inoculation and the reproduction rate of the populations/isolates. Conversely, SFs give an indication of the aggressiveness of the second generation of the invasive J2 hatched in the soil, as well as the level of root galling and plant damage caused by the populations/isolates.

### 3.4. 5-mdC ELISA-based Immunoassays

Roots from Rossol and RomaVF tomato cvs, un-inoculated and inoculated with the field population/virulent isolate couples (*Mi-Vfield/SM2V, Mj-TunC2field/SM11C2*) were used at the 7th day after inoculation to extract total DNA using a specificextraction kit, according to the instructions of the manufacturer (DNA-easy Plant Mini, Qiagen, Hilden, Germany).The relative levels of total DNA methylation between healthy and infested roots were compared using the 5-mdC DNA ELISA kit D5325, according to the manufacturer’s instruction (Zymo Research Corporation, Irvine, CA, USA). DNA aliquots (100 ng) were denaturated and incubated with a mix consisting of anti-5-deoxy-methylcytosine (5-mC) and secondary (horseradish peroxidase conjugate) antibodies. After incubation, these mixtures were added to ELISA plates. Percentages of methylated DNA could be measured by reading the absorbance in an ELISA plate reader at 450 nm. A standard curve of absorbance at 450 nm, as a function of known percentages of 5-mC, had previously to be plotted. The 5-mC amounts of unknown samples could be calculated by a complex equation derived from the logarithmic second-order regression standard curve. Negative control readings were subtracted from the readings of the sample and the standard. The reported values are the means of the absorbance taken at 45 and 60 min since the start of the reactions. Technical duplicated or triplicate DNA samples were obtained from three independent biological assays.

### 3.5. RNA extRaction, cDNA Synthesis, and Quantitative Real-Time Polymerase Chain Reaction 

RNA isolation was carried out from the roots of susceptible (Roma VF) and resistant (Rossol) tomato plants un-inoculated and inoculated with the field population/virulent isolate couple (*Mi-Vfield/SM2V*) at the 7th DAI. RNA was isolated using an RNA-easy Plant Mini Kit according to the instructions specified by the manufacturer (Qiagen, Hilden, Germany). The isolated RNA was loaded on a 1.0% agarose gel and subjected to an electrophoresis run to test its quality; afterwards, it was quantified in a Nano-drop spectrophotometer.cDNAswere synthesized from the isolated RNA using the QuantiTect Reverse Transcripton Kit (Qiagen, Hilden, Germany). qRT-PCR was carried out with the SYBR Select Master Mix (Applied Biosystems Inc., Foster City, CA, USA) according to supplier’s indications, using an Applied Biosystems1 StepOne™ instrument. PCR amplifications were carried out through an initial and final denaturation step at 95 °C (10 min) with 40 intermediate cycles at 95 °C (30 s), 58 °C (30 s), and 72 °C (30 s).The following genes were tested: *cytosine-5 DNA methyl-transferase 1* (NM_001247819.3**,** *Met1*)*, chromo methyl-transferase 2* (NM_001366667.1**,** *CMT2*), and *domains rearranged methyl-transferase 5* (NM_001246974.3**,** *DRM5*). The oligonucleotide primers for each gene are described in Table S1. For each oligonucleotide set, a no-template water control was used. *Actin-7* (NM_001308447.1, *ACT*) was used as the reference gene for quantification, as it was experimented to be the most suitable one for the experimental conditions used in this work. The threshold cycle numbers (C_t_) for each transcript quantification were examined and the relative fold changes in gene expression between infected and uninfected roots were calculated by the 2^-∆∆CT^ method [41].

### 3.6. Protein Extraction and Enzyme Activity Assays

Proteins were extracted from roots and leaves of un-inoculated and inoculated plants1and 5 DAI, and 1 DAI, respectively. Roots and leaves were separated from shoots. Samples of tissues from each RKN-tomato interaction to be used for protein extractions were collected, dried, and weighed. Some samples were immediately ground in porcelain mortars by immersion in liquid nitrogen. Other samples were put on ice and temporarily stored at −80°C. A grinding buffer (1:5 *w:v*) of 0.1 M K-phosphate buffer (pH 6.0), 4% poly-vinyl-pyrrolidone and the protease inhibitor phenyl-methane-sulfonyl fluoride (PMSF, 1 mM)was used to suspend the powdered tissue samples for further grinding by using a Polytron 1 PT–10–35 (Kinematica GmbH, Switzerland). Coarse suspensions were filtered through four layers of gauze and filtrates centrifugedat 12000×*g* for 15 min. Supernatants were collected in 10-ml syringes and filtered through 0.45 μm nitrocellulose filters. An additional ultra-filtration of the supernatants was carried out at 4°C through 20-ml Vivaspin micro-concentrators (10,000 molecular weight cut off, Biotech GmbH, Nordost, Germany). Retained protein suspensions were used as samples for enzyme activity evaluation. Protein content was determined to express specific enzyme activities; the enhanced alkaline copper protein assay was used, with bovine serum albumin, as the standard [42].

Superoxide Dismutase activity (SOD) was determined as the amount of inhibition that the assayed protein suspensions (25–50 µL) caused on the reduction of cytochrome *c* (80 µM) by the xanthine (1 mM)-xanthine oxidase (20 mU) system performed in a standard reaction without plant extracts. One ml assay medium contained 0.1 M Na-K-phosphate buffer (pH 7.8), 20 mM NaN_3_, and 1 mM EDTA. Addition of xanthine oxidase started the reactions, which were monitored at 550/540 nm, in a 557 Perkin–Elmer double-beam spectrophotometer; 50% inhibition on standard reaction represented 1 unit of SOD [43]. Catalase activity (CAT) was detected as the initial rate of disappearance of H_2_O_2_, which provoked a decrease in the absorbance at 240 nm [44]. Reaction mixture (0.5 ml final volume) consisted in 20 mM H_2_O_2_, 25-50 ml tissue extracts, and 0.1 M Na-phosphate buffer, pH 7.0; the oxidation of 1 mmole H_2_O_2_ min^-1^ (*e* = 0.038 mM^-1^ cm^-1^) represented one unit of enzyme. Ascorbate peroxidase activity (APX) was determined as the rate of oxidation of ascorbate by H_2_O_2_ and monitored as a decrease in absorbance at 298 nm [45]. Reaction mixtures (0.5 ml final volume) contained 0.1MTES, pH 7.0, 10–20 μL tissue extracts, 0.1 mM EDTA, 1 mM ascorbate, and 0.1 mM H_2_O_2_; 1 unit of enzyme expressed the oxidation of1 μmole ascorbate min^-1^ (ε = 0.8 mM^-1^ cm^-1^).

The amount of glucose released from laminarin (Sigma-Aldrich S.r.l., Milan, Italy) used as substrate was determined for β-1,3-Endoglucanase activity (GLU) assays. Reaction mixtures consisted in 100μL tissue extracts, 300 μL 0.1M Na-acetate buffer (pH 5.2), and laminarin (0.4 mg); reaction mixtures in plastic eppendorfs were then incubated at 37°C for 30 min. Afterwards, Nelson alkaline copper reagent (300 μL) was added and the mixtures kept at 100°C for 10 min and let to coolat room temperature. Once cooled, mixtures were added with Nelson chromogenic reagent (100 μL) for reducing sugars assays [46]. Grinding buffer and laminarinase (2 U/ml) were used to produce negative and positive controls, respectively. The absorbance at 500 nm of the glucose solutions was compared with the ones of a standard curve created with known amounts (10–200 μg ml^-1^) of commercial glucose (Sigma-Aldrich S.r.l., Milan, Italy). GLU was expressed as μmol glucose equivalents released min^-1^.

The detection of N-acetyl-D-glucosamine (NAG) by a colorimetric procedure was used for chitinase activity (CHI) bioassay [47]. NAG is detected by the β-glucuronidase introduced in the reaction mixture; NAG originates from chetobiose, a product ofthe hydrolytic action of chitinase on chitin. Reaction mixtures contained suspended chitin (250 μL, 10 mgml^-1^) from shrimp shells (Sigma-Aldrich S.r.l., Milam,, Italy) in a Na-acetate buffer (150 μL, 0.05M, pH 5.2) containing 0.5 M NaCl. Such mixtures were incubated for 1 h at the most suitable temperature of 37 °C in an orbital incubator. The reaction was stopped by protein denaturation at 100 °C for 5 min in a water bath. Afterwards, mixtures were centrifuged at 10,000× *g* for 5 min at room temperature and supernatants (300 μL) collected and added with 5 μl β-glucuronidase (Sigma-Aldrich S.r.l., Milan, Italy, type HP-2S, 9.8 units/ml) to produce NAG. Reactions were performed, as previously described. At the end of the reactions, 0.8M K-tetraborate (60 μL, pH 9.1) was added in the mixtures, which were again heated to 100 °C for 3 min and cooled to room temperature. Then, after adding 1% 4-dimethylaminobenzaldehyde(1.2 mL, DMAB, Sigma), the mixtures were incubated at 37 °C for 20 min. Absorbance of the unknown NAG solutions was read at 585 nm (DU-70, Beckman Coulter Life Science, Italy), and amounts calculated by means ofa standard curve obtained with known concentrations (4.5–90 nmoles) of commercial NAG (Sigma). Unspecific absorbance from reactions without tissue extracts (negative controls) was constantly subtracted from sample absorbance; positive controls were arranged by adding 10 μL chitinase from *Streptomyces griseus* (Sigma-Aldrich S.r.l., Milan Italy, 200 units/g). One unit of CHI was expressed as 1.0nmol NAG produced per second at 37 °C. All the enzyme activities were expressed as Units mg^-1^ protein.

### 3.7. Experimental Design and Statistical Analysis

Experiments to test the infection level were designed to use 6 plants for each of the 8 tested RKN-tomato interactions, coming from 2 tomato cvs (Roma VF and Rossol) infected by 4 nematode samples (*Mi-Vfield/SM2V, Mj-TunC2field/SM11C2*). Three subsequent experiments were carried out. Three replications per experiment were arranged; values of infection factors are expressed as means (*n* = 9) ± standard deviation. Means of each tested infection factor were separated by a Duncan’s test (Significance Level: 0.05) carried out by the X-Stat software.

DNA extractions were carried out from bunches of roots from un-inoculated and inoculated plants (6 resistant and 6 susceptible) by 4 nematode samples (*Mi-Vfield/SM2V, Mj-TunC2field/SM11C2*). Two DNA extractions were performed from 2 bioassays. Each DNA sample had 3 replicate readings. Values were expressed as means (*n* = 6) ± standard deviations. Means were separated by a Duncan’s test (Significance Level: 0.05) carried out by the X-Stat software.

RNA extractions were carried out from single susceptible and resistant roots, un-inoculated or inoculated with the field population/virulent isolate couple *Mi-Vfield/SM2V.* There were three extractions per bioassay from 2 bioassays analyzed for gene expression. qRT-PCR data are expressed as means (*n* = 6) ± standard deviations of 2^-∆∆Ct^ values of each group from inoculated plants, considering as 1 the values of each group from un-inoculated plants; significant difference with respect to the un-inoculated controls was determined by a *t-*test (*p < 0.05; **p < 0.01).

Protein extractions were carried out from mixed tissues coming from 2 un-inoculated or inoculated plants, in order to have 3 extractions per experiment. From each of the 3 protein extracts, one value of enzyme activity was determined by 3 technical replicates at the spectrophotometer. Three bioassays were performed in order to have 9 values for each enzyme activity. Means ± standard deviations were calculated out of these values. Means of the un-inoculated controls were separated from those of inoculated plants by a *t-*test (*p < 0.05; **p < 0.01).

## Supplementary Materials

Supplementary materials can be found at https://www.mdpi.com/1422-0067/21/20/7759/s1.

## References

[1] El-Sappah A.H., Islam M.M., El-Awady H.H., Yan S., Qi S., Liu J., Cheng G.-T., Liang Y. (2019). Tomato Natural Resistance Genes in Controlling the Root-Knot Nematode. Genes.

[2] Rossi M., Goggin F.L., Milligan S.B., Kaloshian I., Ullman D.E., Williamson V.M. (1998). The nematode resistance gene Mi of tomato confers resistance against the potato aphid. Proc. Natl. Acad. Sci. USA.

[3] Nombela G., Williamson V.M., Muñiz M. (2003). The Root-Knot Nematode Resistance Gene Mi-1.2 of Tomato Is Responsible for Resistance Against the Whitefly Bemisia tabaci. Mol. Plant-Microbe Interact..

[4] Branch C., Hwang C.-F., Navarre D.A., Williamson V.M. (2004). Salicylic Acid Is Part of the Mi-1-Mediated Defense Response to Root-Knot Nematode in Tomato. Mol. Plant-Microbe Interact..

[5] Molinari S. (2007). New developments in understanding the role of salicylic acid in plant defence. CAB Rev. Perspect. Agric. Vet. Sci. Nutr. Nat. Resour..

[6] Molinari S., Fanelli E., Leonetti P. (2013). Expression of tomato salicylic acid (SA)-responsive pathogenesis-related genes in Mi-1-mediated and SA-induced resistance to root-knot nematodes. Mol. Plant Pathol..

[7] Glazebrook J. (2005). Contrasting Mechanisms of Defense Against Biotrophic and Necrotrophic Pathogens. Annu. Rev. Phytopathol..

[8] Molinari S., Zacheo G., Bleve-Zacheo T. (1990). Effects of nematode infestation on mitochondria isolated from susceptible and resistant tomato roots. Physiol. Mol. Plant Pathol..

[9] Cramer C.L., Weissenborn D., Cottingham C.K., Denbow C.J., Eisenback J.D., Radin D.N., Yu X. (1993). Regulation of Defense-related Gene Expression during Plant-Pathogen Interactions. J. Nematol..

[10] Spoel S.H., Dong X. (2012). How do plants achieve immunity? Defence without specialized immune cells. Nat. Rev. Immunol..

[11] Mendy B., Wang’Ombe M.W., Radakovic Z.S., Holbein J., Ilyas M., Chopra D., Holton N., Zipfel C., Grundler F.M.W., Siddique S. (2017). Arabidopsis leucine-rich repeat receptor–like kinase NILR1 is required for induction of innate immunity to parasitic nematodes. PLoS Pathog..

[12] Zipfel C., Oldroyd G.E.D. (2017). Plant signalling in symbiosis and immunity. Nat. Cell Biol..

[13] Jaouannet M., Magliano M., Arguel M.J., Gourgues M., Evangelisti E., Abad P., Rosso M.-N. (2013). The Root-Knot Nematode Calreticulin Mi-CRT Is a Key Effector in Plant Defense Suppression. Mol. Plant-Microbe Interact..

[14] Xie J., Li S., Mo C., Wang G., Xiao X., Xiao Y. (2016). A Novel Meloidogyne incognita Effector Misp12 Suppresses Plant Defense Response at Latter Stages of Nematode Parasitism. Front. Plant Sci..

[15] Vieira P., Gleason C. (2019). Plant-parasitic nematode effectors—Insights into their diversity and new tools for their identification. Curr. Opin. Plant Biol..

[16] Hewezi T., Baum T.J. (2015). Gene Silencing in Nematode Feeding Sites. Adv. Bot. Res..

[17] Molinari S., Leonetti P. (2019). Bio-control agents activate plant immune response and prime susceptible tomato against root-knot nematodes. PLoS ONE.

[18] Paulson R.E., Webster J.M. (1972). Ultrastructure of the hypersensitive reaction in roots of tomato, Lycopersicon esculentum L., to infection by the root-knot nematode, Meloidogyne incognita. Physiol. Plant Pathol..

[19] Derksen H., Rampitsch C., Daayf F. (2013). Signaling cross-talk in plant disease resistance. Plant Sci..

[20] Tirnaz S., Batley J. (2019). DNA Methylation: Toward Crop Disease Resistance Improvement. Trends Plant Sci..

[21] Alonso C., Ramos-Cruz D., Becker C. (2018). The role of plant epigenetics in biotic interactions. New Phytol..

[22] Elhamamsy A.R. (2016). DNA methylation dynamics in plants and mammals: Overview of regulation and dysregulation. Cell Biochem. Funct..

[23] Atighi M.R., Verstraeten B., De Meyer T., Kyndt T. (2020). Genome-wide DNA hypo-methylation shapes nematode pattern-triggered immunity in plants. New Phytol..

[24] Gheysen G., Mitchum M.G. (2018). Phytoparasitic Nematode Control of Plant Hormone Pathways. Plant Physiol..

[25] Molinari S. (2010). Natural genetic and induced plant resistance, as a control strategy to plant-parasitic nematodes alternative to pesticides. Plant Cell Rep..

[26] Trudgill D.L. (1991). Resistance to and tolerance of plant parasitic nematodes in plants. Annu. Rev. Phytopathol..

[27] Molinari S. (2011). New approach of rating pathogen-host suitability between (a)-virulent populations of root-knot nematodes and tomato. Acta Hortic..

[28] Ammati M., Thomason I.J., Roberts P.A. (1985). Screening Lycopersicon spp. for new genes imparting resistance to root-knot nematodes (Meloidogyne spp.). Plant Dis..

[29] Tirumalaraju S.V., Jain M., Gallo M. (2011). Differential gene expression in roots of nematode-resistant and -susceptible peanut (Arachis hypogea) cultivars in response to early stages of peanut root-knot nematode (Meloidogyn earenaria) parasitization. J. Plant Physiol..

[30] Bali S., Vining K., Gleason C., Majtahedi H., Brown C.R., Sathuvalli V.R. (2019). Transcriptome profiling of resistance response to Meloidogyne chitwoodi introgressed from wild species Solanum bulbocastanum into cultivated potato. BMC Genom..

[31] Jones P.A. (2012). Functions of DNA methylation: Islands, start sites, gene bodies and beyond. Nat. Rev. Genet..

[32] Molinari S., Loffredo E. (2006). The role of salicylic acid in defense response of tomato to root-knot nematodes. Physiol. Mol. Plant Pathol..

[33] Vasyukova N.I., Zinov’Eva S.V., Udalova Z.V., Panina Y.S., Ozeretskovskaya O.L., Sonin M.D. (2003). The role of salicylic acid in systemic resistance of tomato to nematodes. Dokl. Biol. Sci..

[34] Durrant W., Dong X. (2004). Systemic acquired resistance. Annu. Rev. Phytopathol..

[35] Shirasu K., Nakajima H., Rajasekhar V.K., Dixon R.A., Lamb C. (1997). Salicylic Acid Potentiates an Agonist-Dependent Gain Control That Amplifies Pathogen Signals in the Activation of Defense Mechanisms. Plant Cell.

[36] Lin B., Zhuo K., Chen S., Hu L., Sun L., Wang X., Zhang L., Liao J. (2015). A novel nematode effector suppresses plant immunity by activating host reactive oxygen species-scavenging system. New Phytol..

[37] Qtu J., Hallmann J., Kokalis-Burelle N., Weaver D.B., Rodríguez-Kábana R., Tuzun S. (1997). Activity and Differential Induction of Chitinase Isozymes in Soybean Cultivars Resistant or Susceptible to Root-knot Nematodes. J. Nematol..

[38] Molinari S., Caradonna S. (2003). Reproduction of natural and selected resistance-breaking Meloidogyne populations on near-isogenic tomato lines. Nematol. Mediterr..

[39] Williamson V.M., Lambert K.N., Kaloshian I. (1994). Molecular biology of nematode resistance in tomato. Advances in Molecular Plant Nematology.

[40] Molinari S., Narwal S.S., Sampietro D.A., Catalàn C.A.N., Vattuone M.A., Politycka B. (2009). Bioassays on plant–nematode interactions. Plant Bioassays.

[41] Livak K.J., Schmittgen T.D. (2001). Analysis of relative gene expression data using real-time quantitative PCR and the 2-∆∆Ct method. Methods.

[42] Lowry O.H., Rosebrough N.J., Farr A.L., Randall R.J. (1951). Protein measurement with the Folin phenol reagent. J. Biol. Chem..

[43] Furusawa I., Tanaka K., Thanutong P., Mizuguchi A., Yazaki M., Asada K. (1984). Paraquat resistant tobacco calluses with enhanced superoxide dismutase activity. Plant Cell Physiol..

[44] Chance B., Mahley A.C., Colowick S.P., Kaplan N.O. (1955). Assay of catalases and peroxidases. Methods in Enzymology.

[45] Gerbling K.-P., Kelly G.J., Fischer K.-H., Latzko E. (1984). Partial Purification and Properties of Soluble Ascorbate Peroxidases From Pea Leaves. J. Plant Physiol..

[46] Ashwell G. (1957). Colorimetric analysis of sugars. Methods Enzymol..

[47] Reissig J.L., Stromenger J.L., Leloir L.F. (1955). A modified colometric method for the estimation of N-acetyl-amino sugars. J. Biol. Chem..

