# Supplementary material for: Epigenetic and Metabolic Changes in Root-Knot Nematode-Plant Interactions
Source: Int J Mol Sci. 2020 Oct 20;21(20):7759. doi: 10.3390/ijms21207759 (PMC7589425; doi:10.3390/ijms21207759)
Supplement: Supplementary file 1 [file ijms-21-07759-s001.pdf]

Table 1. Accession number, gene description, acronyms and primer sequences used in qRT-PCR.

Accession number and Gene description	Acronyms and Primer sequence (5′-3′)	
**NM_001247819.3 Cytosine-5 DNA methyl-transferase 1 (MET1), mRNA**	SlMET1-F	TGAAGGATGATGAAGAAC
	SlMET1-R	GTAGAAGAAGGTGTATGAG
NM_001366667.1 Chromo methyl-transferase 2 (CMT2), mRNA	SlCMT2-F	CAGGTGAGGAATGAGTAT
	SlCMT2-R	ATCAAGGAACAAGAGACA
NM_001246974.3 DOMAINS REARRANGED METHYL-TRANSFERASE 5 (DRM5), mRNA	SlDRM5-F	TAAGTAGGACCGATAGATA
	SlDRM5-R	CCAGAGAAGAGTGATAAG
NM_001308447.1 Actin-7 (ACT), mRNA	ACT-F	CAGCAGATGTGGATCTCAAA
	ACT-R	CTGTGGACAATGGAAGGAC
